# Association of NT-proBNP with non-alcoholic fatty liver disease in elderly men with normal BMI and incident heart failure

**DOI:** 10.3389/fcvm.2026.1846048

**Published:** 2026-10-13

**Authors:** Jinling Ma, Jinxin Wang, Xuan Zhou, Xiaoqian Li

**Affiliations:** Department of Geriatric Cardiology, the Second Medical Center and National Clinical Research Center for Geriatric Diseases, Chinese PLA General Hospital, Beijing, China

**Keywords:** BMI, elderly, heart failure, nonalcoholic fatty liver disease, NT-ProBNP

## Abstract

**Background:**

With the increasing burden of lean nonalcoholic fatty liver disease (NAFLD) and heart failure (HF), understanding the relationship between lean NAFLD and NT-proBNP in elderly patients becomes clinically important. This study aimed to identify the risk stratification value of combining NT-proBNP and lean NAFLD in elderly men with incident HF.

**Methods:**

The data from consecutive male patients aged 80 years or older, with incident HF of New York Heart Association class III/IV and a BMI less than 23 kg/m^2^, who had available NT-proBNP measurements within 24 h of admission, were retrospectively analyzed. The clinical outcomes were 180-day all-cause and cardiovascular mortality.

**Results:**

A total of 3,627 patients were included. The levels of NT-proBNP were significantly higher in patients without lean NAFLD compared to those with lean NAFLD. After multivariate logistic regression, lean NAFLD remained independently associated with NT-proBNP category. Moreover, during the 180-day follow-up, Kaplan–Meier curves confirmed that patients with both lean NAFLD and NT-proBNP > 8330pg/mL had significantly higher all-cause and cardiovascular mortality compared to those with NT-proBNP alone.

**Conclusion:**

NT-proBNP levels were higher in patients without lean NAFLD than in those with lean NAFLD. The coexistence of lean NAFLD and NT-proBNP > 8330 pg/mL was associated with increased all-cause and cardiovascular mortality in elderly normal-BMI men with incident HF.

## Introduction

The N-terminal pro-B-type natriuretic peptide (NT-proBNP) level is significantly associated with the diagnosis of heart failure (HF) and its severity (1, 2). It has been reported that relatively lower NT-proBNP levels among obese patients or those with a higher body mass index (BMI) (3–7). The prognostic value of NT-proBNP in acute coronary syndrome patients varies with BMI (8). However, BMI alone is insufficient for evaluating body composition. There is excess adiposity in the majority of patients with preserved ejection fraction heart failure (HFpEF), including those without obese (9). The same BMI does not distinguish individuals with different visceral fat, especially among Asians at lower BMI levels. Nonalcoholic fatty liver disease (NAFLD) accelerates cardiovascular-kidney-metabolic progression to increased mortality (10). Additionally, there were potential sex differences in the impact of central adiposity (11), as well as the relationship between obesity and natriuretic peptides (12).

Increasing prevalence of NAFLD has made it the most common chronic liver condition globally (13). Over one-third of NAFLD in elderly cohort occurred in lean individuals (14). Lean metabolic dysfunction-associated fatty liver disease affects many U.S. adults and is often underdiagnosed (15). They are defined as lean NAFLD with a normal BMI <23 kg/m^2^, especially in Asian populations (16, 17). Individuals with lean NAFLD may exhibit similar or even higher morbidity and mortality in comparison to those with obese NAFLD (13). Moreover, lean NAFLD is not considered metabolically benign (18). These individuals were also more likely to experience acute coronary syndromes and metabolic syndrome (19), and may face a higher risk of all- cause mortality compared with lean individuals without NAFLD (20). The presence of lean NAFLD has been demonstrated to be associated with an elevated risk of cerebrocardiovascular events in older patients (21). The patients with lean metabolic dysfunction-associated steatotic liver disease had greater risks of all-cause and diabetes-specific mortality (22). NAFLD may offer simple and cost-effective tools for risk stratification in patients with myocardial infarction (23). It is imperative that NAFLD-related HFpEF is recognized as a distinct HFpEF phenotype (24). Additionally, low levels of NT-proBNP have been demonstrated to be linked to a higher prevalence of NAFLD (25). Given the growing burden of both lean NAFLD and HF, it is essential to understand the interaction between lean NAFLD and NT-proBNP, as well as their combined influence on prognosis of HF, particularly in older populations. This combined risk assessment may offer superior predictive value over either factor alone.

Concerns are growing regarding cardiovascular risk in individuals with lean NAFLD beyond BMI. Implementing fat distribution assessment into clinical practice remains challenging in elderly patients. It is important to explore the prognostic value and identify risk groups and stratification in older men with incident HF using a combined approach of NT-proBNP and the presence of lean NAFLD. The goal of this study was to assess the relationship between NT-proBNP and lean NAFLD, along with the influence of lean NAFLD on NT-proBNP levels among elderly men with incident HF. We also investigated the incremental prognostic value of NT-proBNP and lean NAFLD for risk stratification and targeted intervention in incident HF.

## Methods

### Study participants

The retrospective and observational study population comprised consecutive patients who underwent abdominal ultrasound at Chinese PLA General Hospital between December 2014 and December 2024. The patients aged 80 years and older, with incident HF of New York Heart Association (NYHA) class III or IV, a BMI less than 23 kg/m^2^, and available NT-proBNP measurements within 24 h of admission were included. Demographic and clinical data were obtained from the medical records. NAFLD was determined based on the presence of hepatic steatosis detected by abdominal ultrasonography. Participants were excluded for the following reasons: viral hepatitis, alcoholic liver disease, drug-induced liver injury, biliary tract disorders, hemochromatosis, autoimmune hepatitis, malignant liver diseases, or other secondary causes of liver-fat accumulation. We further excluded individuals with implausible or extreme values of BMI or weight (BMI < 15.0 kg/m^2^ or weight < 30 kg). Among the included participants, those with NAFLD and a BMI less than 23 kg/m^2^ were classified as having lean NAFLD.

### Clinical outcomes

The primary outcome was the 180-day all-cause mortality after enrollment. The secondary endpoint was cardiovascular mortality within each category. Follow-up information was obtained from electronic medical records or by telephone interviews. Cardiovascular death was defined as death caused by myocardial infarction, HF, or significant arrhythmias. Sudden and unexpected death, absent any further explanation, was also considered cardiovascular death.

### Measurement of plasma NT-proBNP

The measurement of NT-proBNP was conducted via electrochemiluminescence immunoassay using the Dimension Vista 500 Intelligent Laboratory System (Siemens Healthcare Diagnostics, Deerfield, Illinois, USA).

### Statistical analysis

Normally distributed continuous variables were represented as mean ± standard deviation (SD) and analyzed with one-way analysis of variance (ANOVA) for intergroup comparisons. Categorical variables were reported with numbers (percentages) and were tested through chi-square tests. Furthermore, multivariate logistic regression analysis adjusted for history of hypertension, atrial fibrillation (AF), and chronic kidney disease (CKD) were used to assess the independent relationship between NT-proBNP and lean NAFLD. The factors were entered into the logistic regression analysis if they had a univariate *P* value <0.05. Odds ratios (OR) were reported with 95% confidence intervals (CI). Finally, Kaplan – Meier curves with the log-rank test were used to explore the association between NT-proBNP levels, lean NAFLD status, and clinical outcomes. We used SPSS 20 (IBM SPSS Statistics, Armonk, NY) to perform all statistical analyses. A two-sided *P* < 0.05 was considered statistically significant.

## Results

### Characteristics of the study participants

A total of 3,627 participants were enrolled in the analysis. The mean age of the individuals was 86.69 ± 5.05 years. The baseline characteristics of the three groups by NT-proBNP category are presented in Table 1. Individuals in the highest NT-proBNP group were more likely to have the lowest prevalence of lean NAFLD. Furthermore, CKD and AF were more frequent in the higher NT-proBNP group than in the lower group. Additionally, the lowest NT-proBNP group were less likely to have hypertension compared to the highest group. Notably, the prevalence of lean NAFLD significantly decreased with increasing NT-proBNP levels (Figure 1). After multivariate logistic regression adjusted for history of hypertension, AF, and CKD, the presence of lean NAFLD remained independently associated with NT-proBNP category (Figure 2). Figures 3–5 showed NT-proBNP levels according to the lean NAFLD status across the three NT-proBNP groups. NT-proBNP levels were lower in participants with lean NAFLD (*P* < 0.001). The levels of NT-proBNP were higher in absence of lean NAFLD patients compared to those with lean NAFLD.

**Table 1:** Baseline characteristics stratified by NT-proBNP groups.

Characteristic	NT-proBNP <1800pg/mL	1800 pg/mL ≤ NT-proBNP ≤ 8330 pg/mL	NT-proBNP > 8330pg/mL	*P*
	*N* = 416	*N* = 975	*N* = 2236	
Age (year)	86.887 ± 5.155	86.852 ± 5.009	86.579 ± 5.045	0.257
Lean NAFLD, *n* (%)	345 (82.9)	495 (50.8)	608 (27.2)	<0.001
Comorbidities, *n* (%)				
Coronary artery disease	330 (79.3)	789 (80.9)	1858 (83.1)	0.100
Diabetes mellitus	135 (32.5)	312 (32.0)	762 (34.1)	0.476
Hypertension	120 (28.9)	494 (50.7)	1585 (70.9)	<0.001
Atrial fibrillation	34 (8.2)	243 (24.9)	895 (40.0)	<0.001
CKD	43 (10.3)	253 (25.9)	1017 (45.5)	<0.001
Comedications, *n* (%)				
Statins	320 (76.9)	769 (78.9)	1789 (80.0)	0.329
*β*-Blockers	171 (41.1)	429 (44.0)	1029 (46.0)	0.144
CCBs	177 (42.5)	409 (41.9)	985 (44.0)	0.513
ACE inhibitors/ARBs	232 (55.8)	507(52.0)	1227(54.9)	0.256

NAFLD, nonalcoholic fatty liver disease; CKD, chronic kidney disease; CCB, calcium channel blocker; ACE, angiotensin converting enzyme inhibitor; ARB, angiotensin receptor blocker.

**Figure 1:**
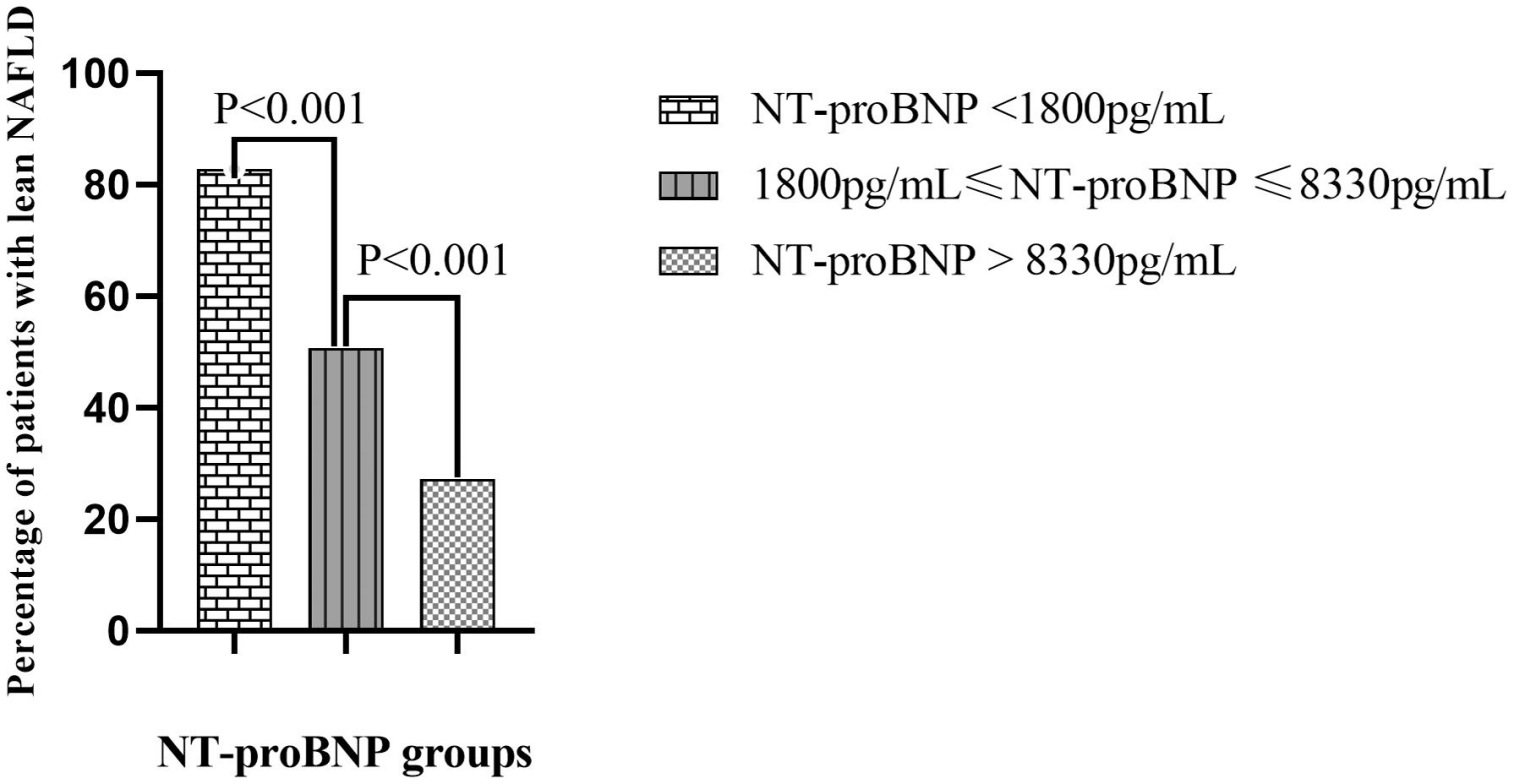
Percentage of patients with lean NAFLD according to NT-proBNP groups.

**Figure 2:**
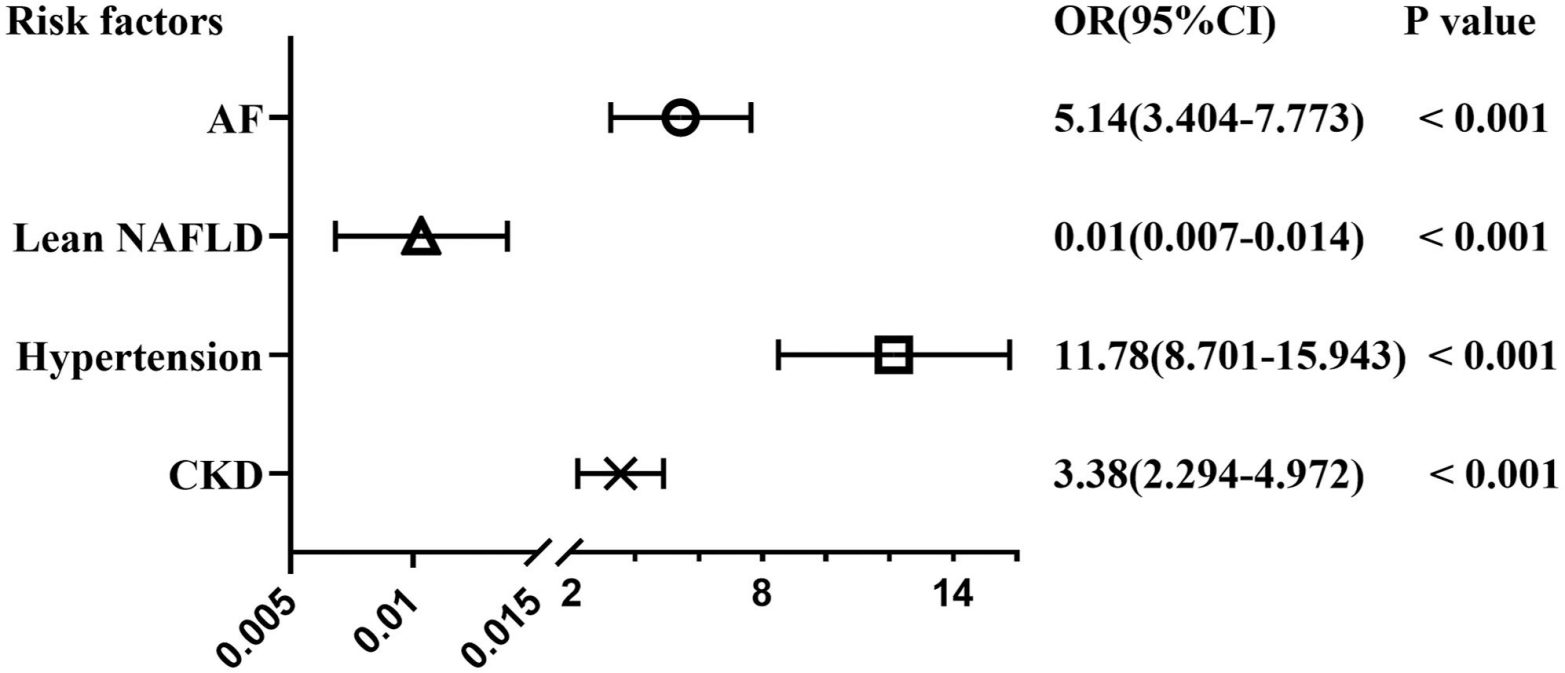
Associations of risk factors with NT-proBNP in the multivariate logistic regression analysis.

**Figure 3:**
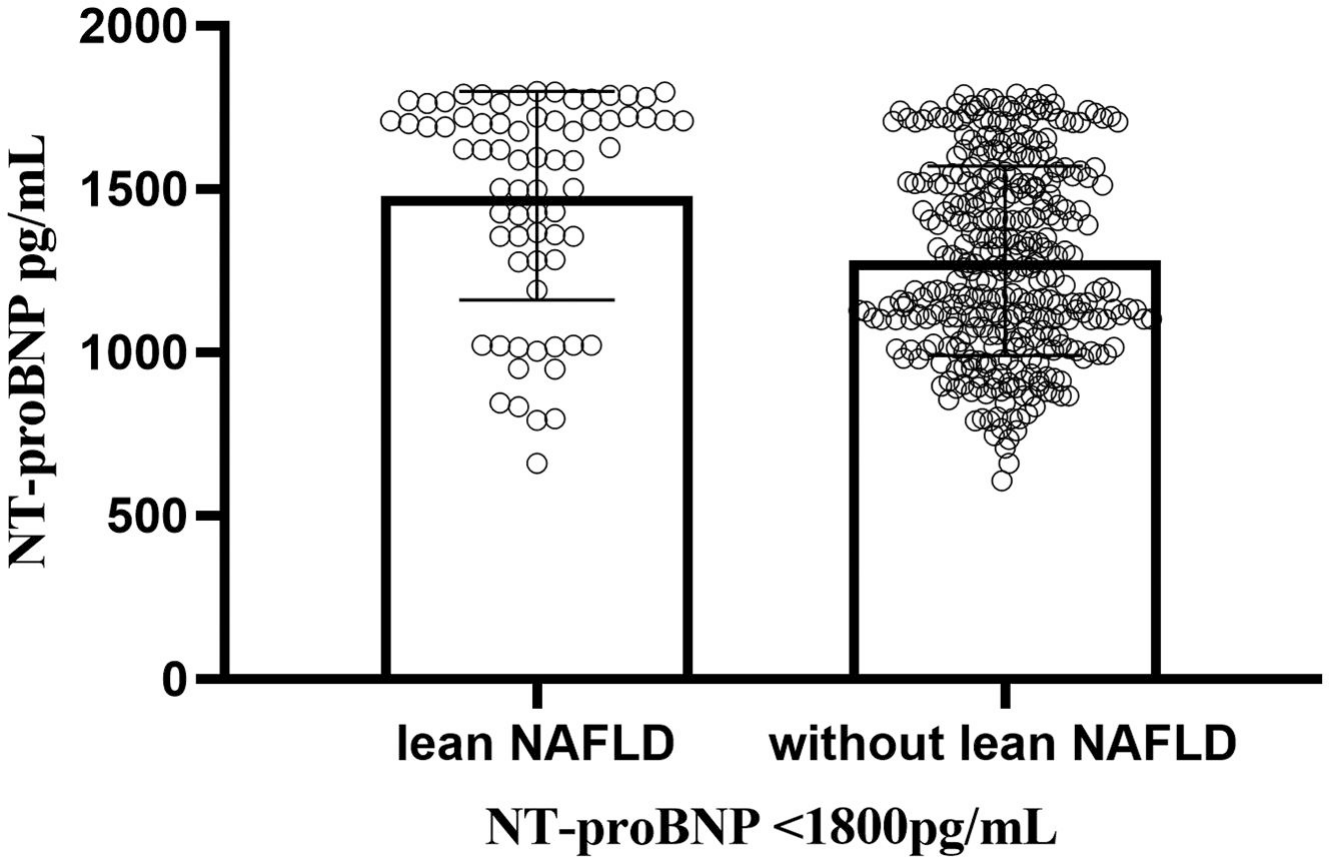
The levels of NT-proBNP < 1800pg/mL with lean NAFLD.

**Figure 4:**
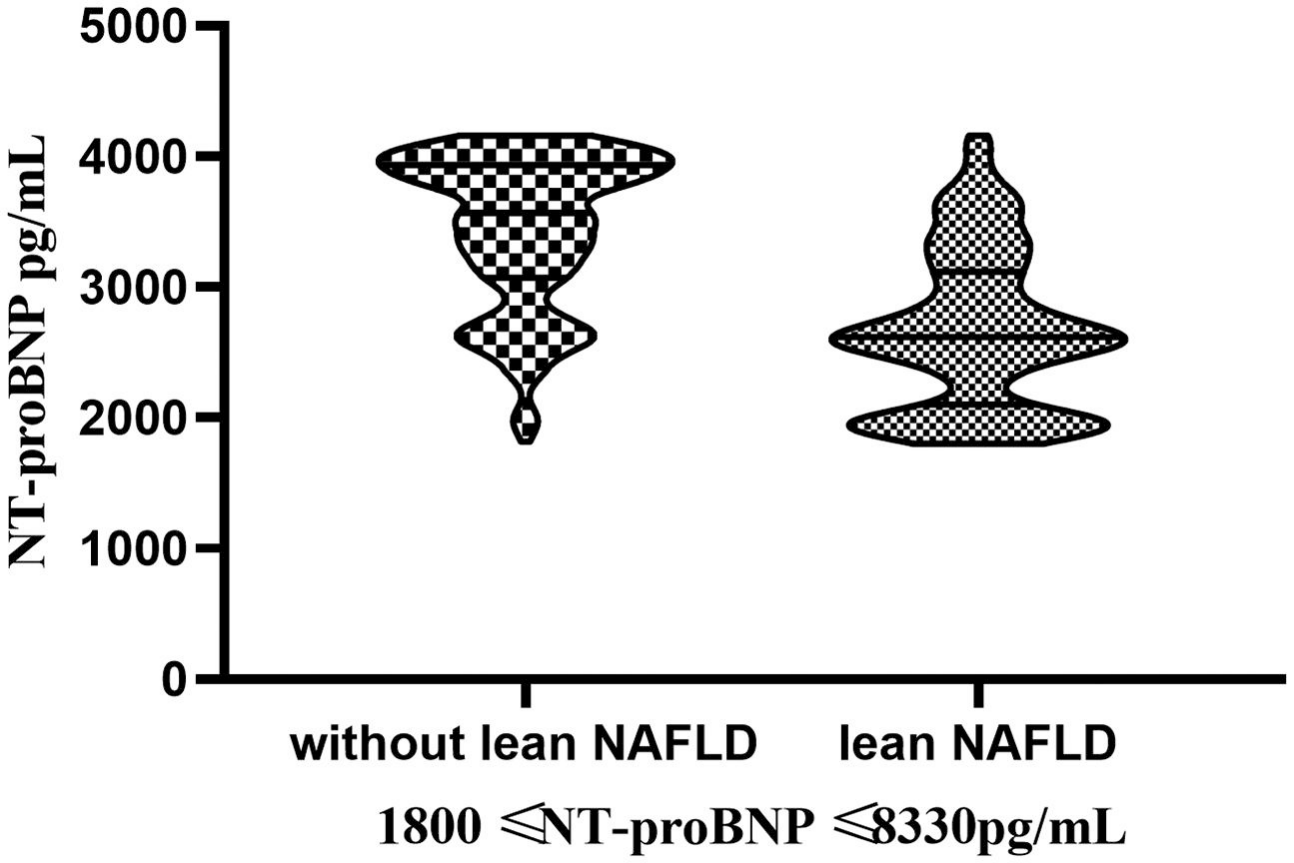
The levels of 1800 ≤ NT-proBNP ≤ 8330pg/mL with lean NAFLD.

**Figure 5:**
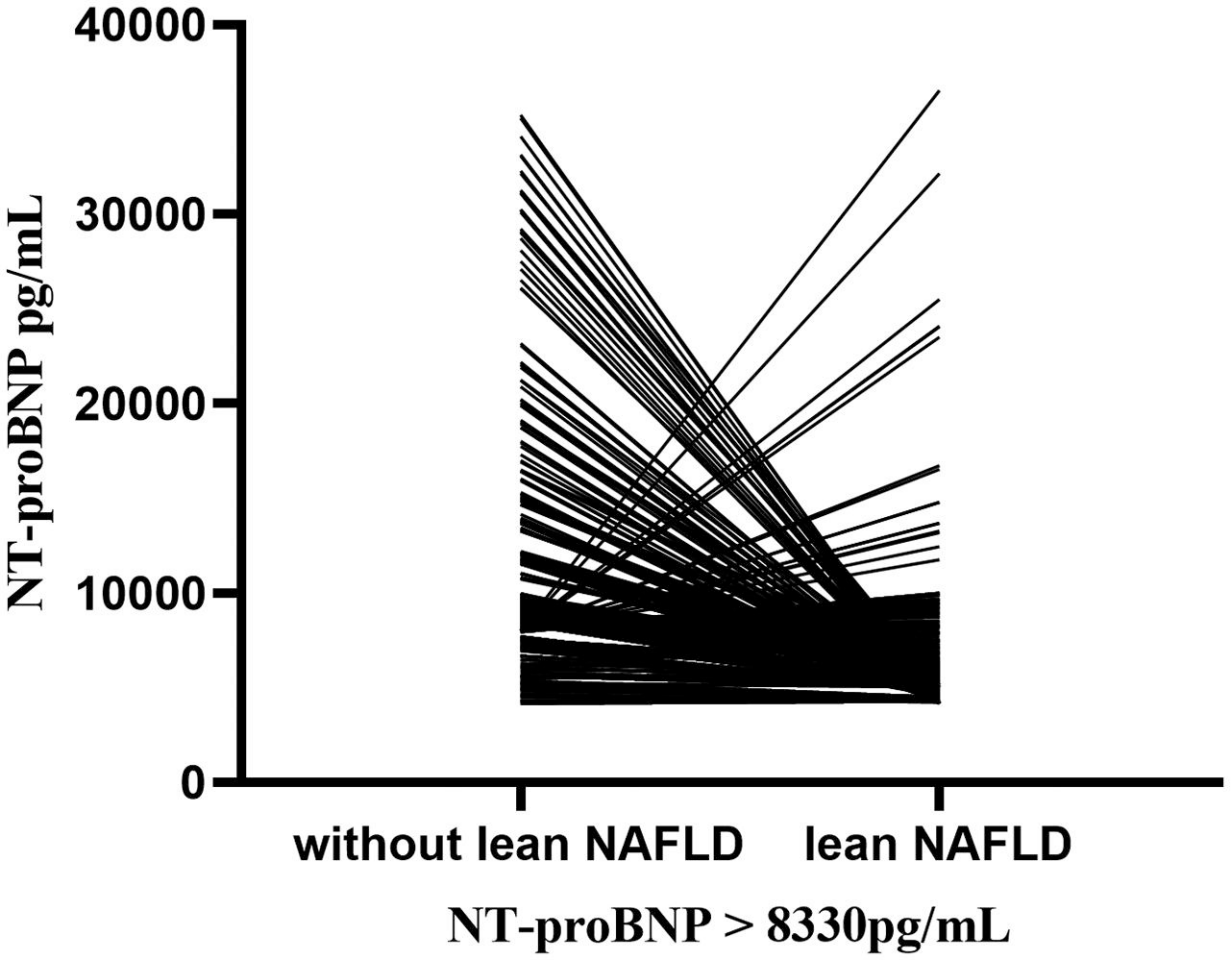
The levels of NT-proBNP > 8330pg/mL with the lean NAFLD.

### Clinical outcomes

During 180-day follow-up, all-cause death occurred in 1,211 participants. In addition, 1,115 participants experienced cardiovascular death. The highest level of NT-proBNP was significantly associated with an increased risk of cardiovascular and all-cause mortality (Table 2). Similarly, the cumulative incidence of all-cause and cardiovascular mortality was higher in patients with NT-proBNP > 8330pg/mL compared to those with lower NT-proBNP levels among individuals with lean NAFLD. Moreover, higher all-cause and cardiovascular mortality was observed in patients with lean NAFLD than in those without lean NAFLD (*P* < 0.001; Table 3).

**Table 2:** Clinical outcomes stratified by NT-proBNP groups during 180-day follow-up.

Clinical outcomes	NT-proBNP <1800pg/mL	1800 pg/mL ≤ NT-proBNP ≤8330 pg/mL	NT-proBNP > 8330 pg/mL	*P*
	*N* = 416	*N* = 975	*N* = 2236	
All-cause death, *n* (%)	53 (12.7)	214 (21.9)	944 (42.2)	<0.001
Lean NAFLD, *n* (%)	33 (7.9)	116 (11.9)	532 (23.8)	<0.001
Cardiovascular death, *n* (%)	45 (10.8)	180 (18.5)	890 (39.8)	<0.001
Lean NAFLD, *n* (%)	30 (7.2)	101 (10.4)	519 (23.2)	<0.001

NAFLD, nonalcoholic fatty liver disease.

**Table 3:** Clinical outcomes stratified by lean NAFLD status during 180-day follow up.

Mortality	Lean NAFLD	Without lean NAFLD	*P*
	*N* = 1448	*N* = 2179	
All-cause, *n* (%)	681 (47.0)	530 (24.3)	<0.001
Cardiovascular, *n* (%)	650 (44.9)	465 (21.3)	<0.001

NAFLD, nonalcoholic fatty liver disease.

The Kaplan – Meier survival curves confirmed the significantly different prognostic value of NT-proBNP in cardiovascular and all-cause mortality according to lean NAFLD status. The Kaplan – Meier curves showed that patients with both lean NAFLD and NT-proBNP > 8330pg/mL had higher all-cause and cardiovascular mortality rates than those with high NT-proBNP alone (*P* < 0.001; Figures 6, 7). Moreover, among individuals with lean NAFLD, those with NT- proBNP > 8330pg/mL had a higher risk of adverse outcomes (cardiovascular and all-cause mortality) compared with those of lower NT-proBNP levels (Figures 8, 9).

**Figure 6:**
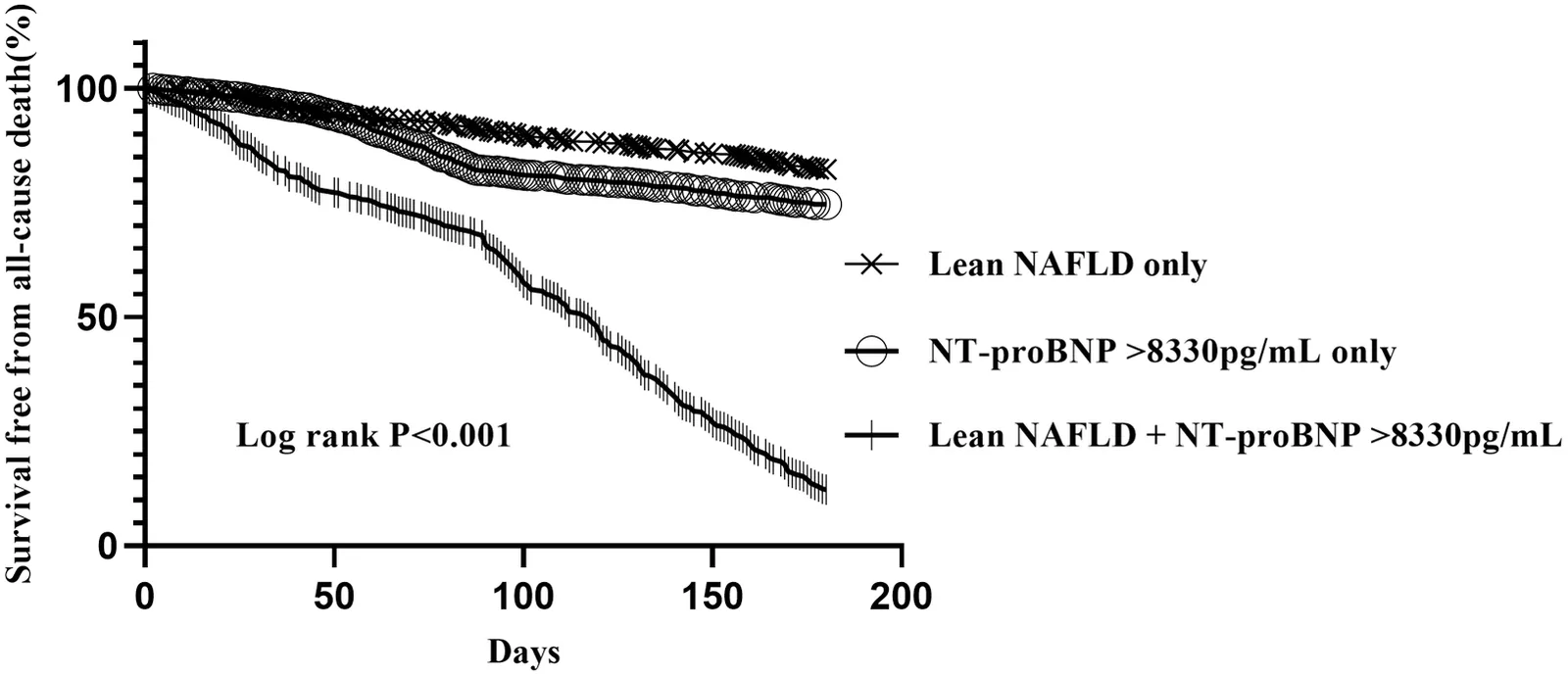
Kaplan–Meier all-cause mortality event-free survival curves according to the NT-proBNP levels and lean NAFLD.

**Figure 7:**
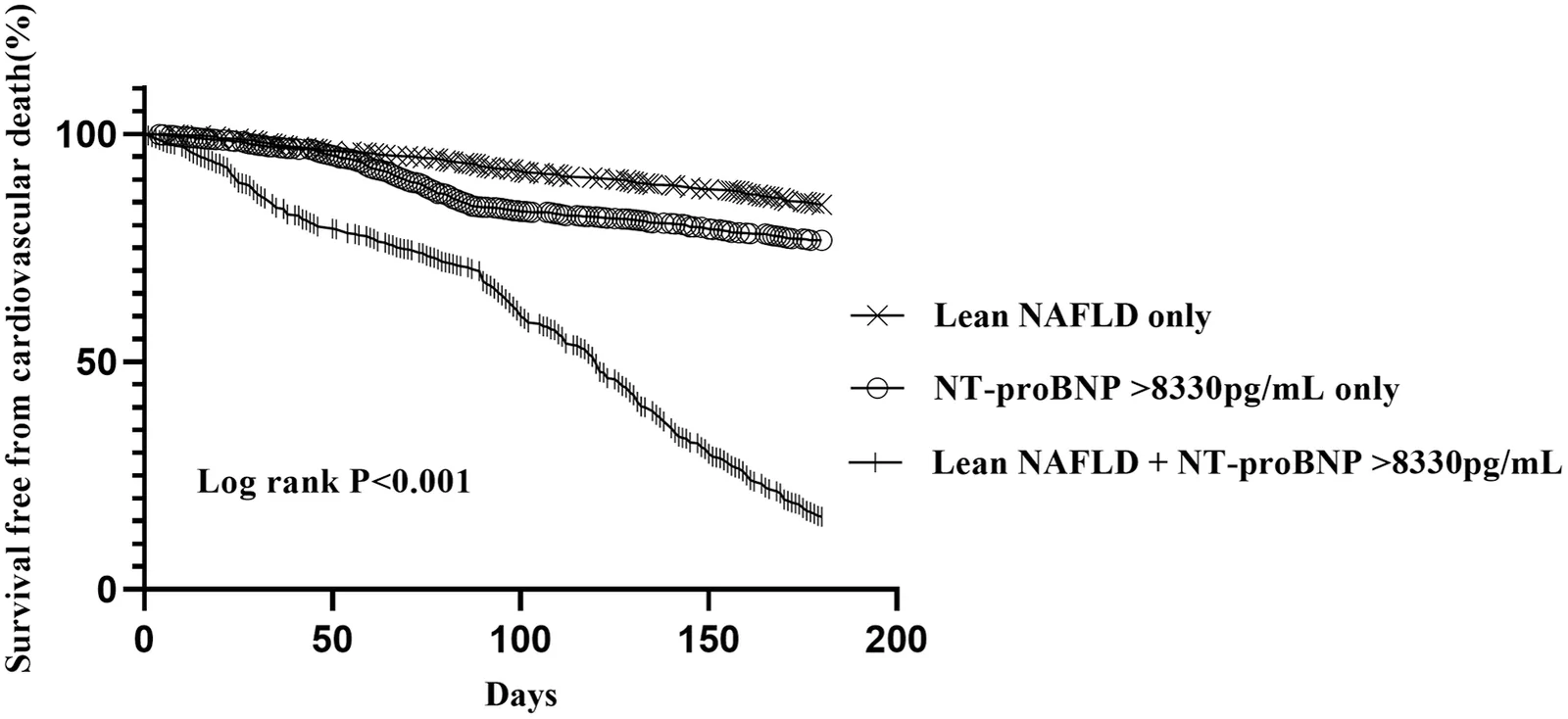
Kaplan–Meier cardiovascular mortality event-free survival curves according to the NT-proBNP levels and lean NAFLD.

**Figure 8:**
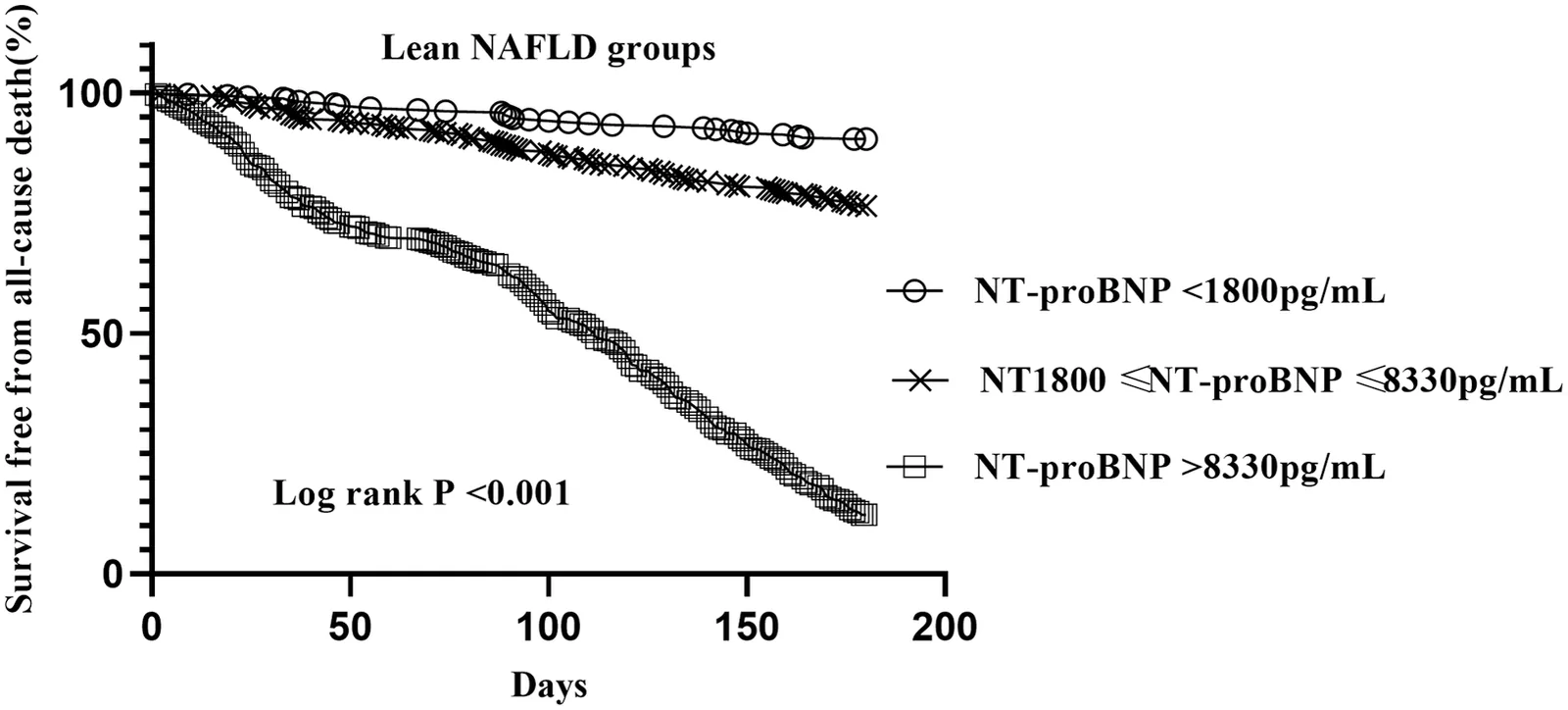
Kaplan–Meier all-cause mortality event-free survival curves according to the NT-proBNP levels among lean NAFLD men.

**Figure 9:**
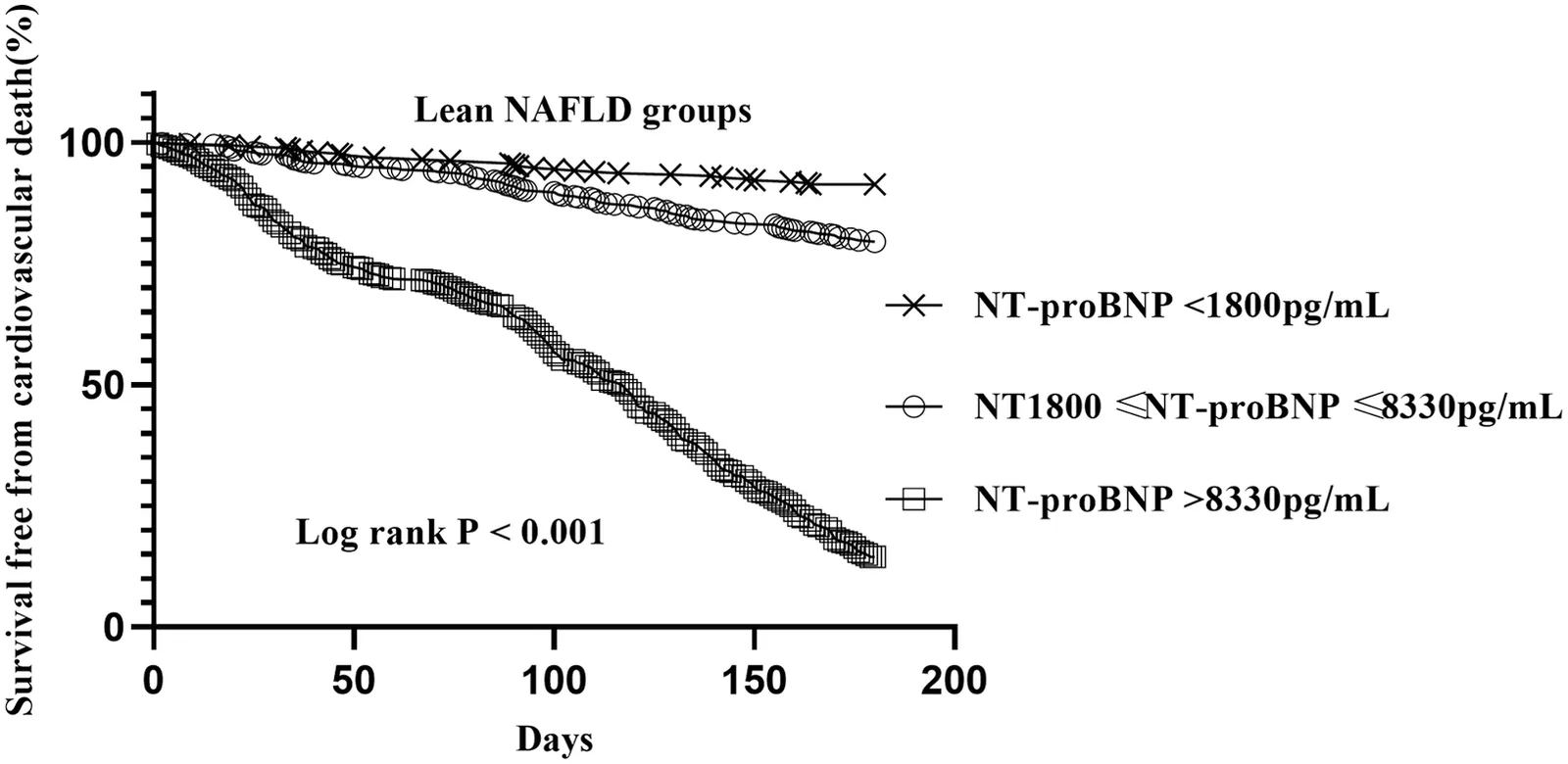
Kaplan–Meier cardiovascular mortality event-free survival curves according to the NT-proBNP levels among lean NAFLD men.

## Discussion

In the present study, individuals with lean NAFLD had significantly lower NT-proBNP levels compared to those without lean NAFLD in elderly, normal BMI male population with incident HF. Furthermore, we found that the male patients in the higher NT-proBNP group had a lower prevalence of lean NAFLD compared to those in the lower NT-proBNP group. Lower NT-proBNP levels were significantly correlated with a higher prevalence of lean NAFLD. Moreover, our data discovered that patients with concurrent lean NAFLD and elevated NT-proBNP levels > 8330 pg/mL had higher 180-day all-cause and cardiovascular mortality rates than those with NT-proBNP alone. We identified 8330pg/ mL as the optimal value for NT-proBNP in elderly men with incident HF, which was based on our experience. This suggests that the combination of both factors may help identify elderly men individuals with normal BMI and incident HF at higher risk of adverse events.

The majority of NAFLD patients demonstrate an elevated risk of experiencing severe cardiovascular events within a decade, irrespective of the degree of liver fibrosis. NAFLD has been demonstrated to be associated with structural and functional cardiac abnormalities commonly observed in patients with HFpEF (26). The prevalence of lean NAFLD in Chinese cohorts is markedly elevated in comparison to that observed in Western populations, despite comparable BMI values (27). In addition, age is a significant factor that must be taken into consideration in lean NAFLD patients, and the prognosis is worse in older individuals. Subjects with lean NAFLD had an increased risk of all-cause mortality (28–30). NAFLD was found to be linked with lower NT-proBNP levels (31). With this growing understanding of the influence of lean NAFLD on NT-proBNP, it is important to understand the potential underlying mechanisms of the inverse relationship between NT-proBNP and lean NAFLD. The reduced levels of NT-proBNP found in obese patients can be attributed to a decreased cardiac production of BNP, as opposed to increased peripheral clearance (32). Furthermore, the correlation between obesity or visceral adipose tissue and cardiovascular disease outcome is also influenced by sex differences (33, 34). NT-proBNP levels in the context of obesity or body composition/mass exhibit sex-based differences (35). Complex and multifactorial mechanisms may contribute to the relationship among NT-proBNP, HF risk, and lean NAFLD.

Our findings are in line with previous studies demonstrating the influence of NT-proBNP on NAFLD patients (31), but the present study only included patients with incident HF of NYHA class III/IV, rather than class I/II. Considering the increasing prevalence of both lean NAFLD and HF in elderly men with incident HF, the implementation of lean NAFLD assessment into the prognostic evaluation using NT-proBNP remains challenging. Early risk stratification in elderly patients with incident HF is very important, and it may help further identify adults who could benefit from the most appropriate therapy as soon as possible, including individualized treatment. In addition, in elderly patients the poor prognosis is possibly influenced by age, more prevalent comorbidity, and underutilization of guideline-recommended therapies (36). Our findings provide additional insight beyond BMI into the risk assessment of clinical outcomes with the interaction relationship between NT-proBNP levels and lean NAFLD. This study also shed light on the prognostic utility of NT-proBNP when incorporating the confounding effect of lean NAFLD in risk stratification, especially in elderly patients with lower NT-proBNP levels than expected. Our results specifically highlight the potential value of lower NT-proBNP levels to improve HF risk prediction in individuals with lean NAFLD. Focusing on NT-proBNP levels, along with lean NAFLD status in normal BMI men, may contribute to improving the prognosis of patients with incident HF.

Several limitations in the present study should be acknowledged. First, this was an observational study with a retrospective design. We did not have information on echocardiography about global longitudinal strain, an important determinant of NT-proBNP levels. Additionally, some important variables such as troponin, lipid profile, frailty score, as well as eGFR were not included. Those potential unmeasured confounding factors could influence the results. There is a possibility that troponin could predict the clinical outcomes similarly or even better than NT-proBNP. The effects should be further investigated. Second, only a single baseline measurement of NT-proBNP was used, without persistent monitoring. Thus, the relationship between changes of NT-proBNP and prognosis was unable to be determined. The change level of NT-proBNP is useful for identifying patients at a high risk of incident HF in elderly type 2 myocardial infarction patients (37). Further data are needed to assess the role of NT-proBNP as a continuous indicator of dynamic changes to guide personalized therapy optimization. Third, all elderly male individuals in our study were from northern China, and thus these findings may not be applicable to other populations or ethnicities, given that body fat distribution patterns vary across geographic regions and race. Also, our results may not be generalizable to younger individuals or female populations. Fourth, abdominal ultrasonography was used to identify NAFLD rather than liver biopsy. Ultrasonography is widely used in clinical practice due to its simplicity and safety, and liver biopsy is often impractical and invasive. Yet ultrasonography cannot assess fibrosis severity. This may lead to underestimates the presence of NAFLD in lean individuals, and there may be a potential for misclassification, which could affect the interpretation. Fifth, we identified 8330pg/ mL of NT-proBNP as the cut-off value. The optimal cut-off for elevated NT-proBNP to provide meaningful predictive information needs validation. Therefore, further investigation of an ROC analysis with the Youden index is required to certify the optimal cut-off value for providing important predictive information. Finally, the 180-day follow-up period was relatively short, and provided short-term prognostic insight. Therefore, it was not possible to determine the effect on long-term results. Future prospective studies should be conducted to further confirm and address these limitations. Further comprehensive investigation is needed to determine the relationships between various demographic factors and NT-proBNP levels in elderly lean NAFLD subjects with incident HF according to sex, age groups and race.

## Conclusion

In conclusion, the assessments of a combined NT-proBNP and lean NAFLD may provide better risk stratification for elderly normal BMI men with incident HF in clinical practice. Consequently, further cohort studies are needed to examine new prediction strategies for identifying the excess burden in elderly male with incident HF who have both lean NAFLD and elevated NT-proBNP, rather than relying solely on BMI.

## Data Availability

The original contributions presented in the study are included in the article/Supplementary Material, further inquiries can be directed to the corresponding author.
